# FIBT versus florbetaben and PiB: a preclinical comparison study with amyloid-PET in transgenic mice

**DOI:** 10.1186/s13550-015-0090-6

**Published:** 2015-03-28

**Authors:** Behrooz H Yousefi, Boris von Reutern, Daniela Scherübl, André Manook, Markus Schwaiger, Timo Grimmer, Gjermund Henriksen, Stefan Förster, Alexander Drzezga, Hans-Jürgen Wester

**Affiliations:** Department of Pharmaceutical Radiochemistry, Technische Universität München, Walther-Meißner-Str. 3, 85748 Garching, Germany; Department of Nuclear Medicine, Technische Universität München, Ismaninger Straße 22, 81675 Munich, Germany; Department of Psychiatry and Psychotherapy, Technische Universität München, Ismaninger Straße 22, 81675 Munich, Germany; Institute of Neuroscience and Medicine (INM-3), Research Centre Jülich, Wilhelm-Johnen-Straße, 52428 Jülich, Germany; Department of Nuclear Medicine, University of Cologne, Kerpener Straße 62, 50937 Cologne, Germany

**Keywords:** Alzheimer’s disease, β-amyloid, Small-animal PET, *In vivo* radiotracers ranking, FIBT, Florbetaben, PiB, APP/PS1 transgenic mouse animal model of AD, *In vivo* imaging, Autoradiography

## Abstract

**Background:**

Over the last decade, an increasing number of studies have been published on the use of amyloid-β (Aβ) PET imaging with different ^18^F-radiopharmaceuticals for clinical characterization of Alzheimer’s disease (AD) in different stages. However, distinct study cohorts and different quantification techniques allow only for an indirect comparison between the different tracers. Thus, the aim of this study was the direct intra-individual *in vivo* comparison of different Aβ-targeted radiopharmaceuticals for PET imaging, including the newly developed agent [^18^F]FIBT.

**Methods:**

A small group of four animals of a well-characterized APP/PS1 transgenic (tg) mouse model of AD and gender-matched control (ctl) animals underwent a sequential and standardized PET imaging regimen for direct comparison of [^18^F]FIBT, [^18^F]florbetaben, and [^11^C]PiB. The quantitative PET imaging data were cross-validated with the cerebral Aβ plaque load as quantified *ex vivo* on histological sections.

**Results:**

We found that FIBT (2-(*p*-methylaminophenyl)-7-(2-[^18^F]fluoroethoxy)imidazo[2,1-*b*]benzothiazole) compares favorably to florbetaben as a high-contrasting PET radiopharmaceutical for imaging Aβ pathology. The excellent pharmacokinetics of FIBT in combination with its high-binding affinity towards Aβ resulted in feasible high-contrast imaging of Aβ with high global cortex to cerebellum standard uptake value ratio (SUVR) in 24-month-old tg mice (tg 1.68 ± 0.15 vs. ctl 0.95 ± 0.02). The SUVRs in transgenic versus control animals (SUVR_tg_/SUVR_ctl_) for FIBT (1.78 ± 0.16) were similar to the ratios as observed in humans (SUVR_AD_/SUVR_ctl_) for the established gold standard Pittsburgh compound B (PiB) (1.65 ± 0.41).

**Conclusions:**

This head-to-head PET tracer comparison study in mice indicated the good imaging properties of [^18^F]FIBT, such as high initial brain uptake, fast clearance of the brain, and high binding affinity towards Aβ as directly compared to the established amyloid tracers. Moreover, the preclinical study design is recommendable for reliable assessment and comparison of novel radiopharmaceuticals.

**Electronic supplementary material:**

The online version of this article (doi:10.1186/s13550-015-0090-6) contains supplementary material, which is available to authorized users.

## Background

Amyloid-β (Aβ) PET has become an important research biomarker for diagnosing and differentiating neurodegenerative disorders, in particular Alzheimer’s disease (AD). In the last decade, major efforts to develop and assess radiopharmaceuticals for early visualization and quantification of Aβ deposits with PET have been reported [1-3]. These endeavors were related to the amyloid cascade hypothesis which assumes that fibrillar Aβ is one of the key pathogenic hallmarks of AD [4-6], therefore, it has been depicted as major target for non-invasive diagnosing of AD. They have resulted in a variety of newly developed radiopharmaceuticals which have been assessed in preclinical and clinical studies [7-10]. Currently, three ^18^F-labeled PET radiopharmaceuticals for Aβ have been evaluated in clinical studies and are now approved by the United States Food and Drug Administration (FDA) and by the European Medicines Agency (EMA): flutemetamol [11,12], florbetaben [13-15], and florbetapir [16-24]. At least two other tracers are already undergoing clinical investigation [25,26]. In our lab, a new series of Aβ ligands, imidazo[2,1-b]benzothiazole (IBT), has been developed inspired by positive imaging results obtained in the IMPY and Pittsburgh compound B (PiB) studies. The IBT structural motifs share features with IMPY and PiB. Furthermore, it also allows stable ^18^F-fluorination. Our aim was to develop a more sensitive Aβ ligand that is easy to synthesize and therefore allows early and practicable detection of amyloid deposition [25,27,28]. With several ^18^F-labeled tracers competing, comparative conclusions are of high interest. However, most of the published studies so far have relied on different methodologies and cohorts, and a direct comparison and ranking in a clinical setting is challenging for their different characteristics [29]. These limitations draw the attention to preclinical approaches for a direct intra-individual *in vivo* comparison of different ^18^F-labeled Aβ tracers.

Alongside tracer development advances subsequent to the application of PiB in a clinical setting [30], Aβ PET imaging was advanced with more sophisticated detection modes and highly sensitive detectors combined with powerful reconstruction and quantification algorithms [31]. In addition, several transgenic animal models of AD have been established for preclinical evaluation of experimental treatments [32-34,10]. Aβ PET imaging in transgenic mouse models of AD is a powerful tool to assess Aβ tracer properties *in vivo* [27,35-38]. However, recent publications made it clear that the type of mouse model seems to play a crucial role as they differ in extent and type of Aβ pathology [39,35]. A new APP/PS1 transgenic mouse model of AD, expresses mutant forms of human amyloid precursor protein (APP) and presenilin-1 (PS1), was characterized [33] in collaboration with our group for its application in Aβ imaging [40] and usage for the evaluation of new Aβ ligands [25,27,40]. For this mouse model, a linear relationship of Aβ plaque pathology and PiB PET signal was observed [41].

**Scheme 1:**
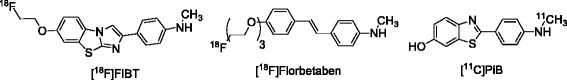
**Chemical structure of the three Aβ radiopharmaceuticals used in this study.**

In the current study, we employed this APP/PS1 mouse model for a direct comparison of two established and one newly developed Aβ PET tracer (Scheme 1). Among the ^18^F-labeled Aβ PET radiopharmaceuticals, [^18^F]florbetaben has shown good sensitivity and specificity for the detection of AD [42]. Hence, [^18^F]florbetaben was selected as reference ^18^F-radiopharmaceutical and compared with a promising in-house developed Aβ PET tracer [^18^F]FIBT [25,27] and the ^11^C-labeled gold standard [^11^C]PiB. *In vivo* small animal PET imaging and *ex vivo* validation experiments were carried out for ranking the tracers.

In order to estimate the translational value of the preclinical results, we compared [^11^C]PiB standard uptake value ratio (SUVR) values from our APP/PS1 and control mice with human [^11^C]PiB SUVR values from 20 AD patients and 15 healthy elderly volunteers.

## Methods

### Animals

Animal experiments were carried out with the approval of the institutional animal care committee (Regierung von Oberbayern, München, Germany) and in accordance with the German Animal Welfare Act. Animal husbandry followed the regulations of the European Union (EU) guideline no. 2010/63. All imaging experiments were performed with male mice: four homozygous APP/PS1 mice ((B6;CB-Tg(Thy1-Psen1*M146V/Thy1-APP*swe)-10Arte), TaconicArtemis GmbH, Cologne, Germany) on a congenic C57BL/6 J genetic background and three age-matched control mice (C57BL/6 J). Mean age and mean weight: 23.8 ± 0.4 months and 33.3 ± 3.2 for APP/PS1 mice (tg) and 24.6 ± 0.4 months and 38.8 ± 2.6 g for C57BL/6 J control mice (ctl), respectively. For biodistribution studies, BALB/c mice were obtained from Charles River Laboratories, Sulzfeld, Germany.

### Human subjects

Human data comes from preexisting studies at the Department of Nuclear Medicine with subjects who were recruited from the outpatient memory clinic of the Department of Psychiatry at the Technische Universität München (TUM). They had been referred for the diagnostic evaluation of cognitive impairment by general practitioners, neurologists, psychiatrists, or from other institutions. They underwent a standardized diagnostic protocol. All examinations were part of their routine check-up in the course of the evaluation of the patient’s neurodegenerative disorders. The psychometric workup was based on the Consortium to Establish a Registry for AD (CERAD) neuropsychological battery, which includes the Mini-Mental-State Examination (MMSE). All patients provided a written informed consent regarding the scientific evaluation of their data.

### Radiosynthesis

All chemicals, solvents, and materials were purchased and directly used without further purification. [^18^F]florbetaben (8% ± 2% radiochemical yield (RCY) and 98% radiochemical purity) [43,44], [^18^F]FIBT (20% ± 4% RCY and 98% radiochemical purity) [25], and [^11^C]PiB (26% ± 5% RCY and 98% radiochemical purity) [45-47,27] were synthesized following already published procedures. Analytical HPLC was performed using either a Chromolith RP-C18e (4.6 × 100 mm; Merck, Dramstadt, Germany) eluted with a mixture of acetonitrile/0.1-M ammonium formate (MeCN content between 27.5% and 50%) at a flow rate of 5 mL/min or a Nucleosil 100, CN 4.6 × 250 mm reverse phase column, particle size 5 μm (CS-Chromatographie, Langerwehe, Germany) eluted with acetonitrile/0.1-M ammonium formate (MeCN content between 40% and 60%). Both chromatography systems were fitted with a UV detector (Sykam Model S3210 set at 254 nm; Sykam, Fürstenfeldbruck, Germany). Radiotracers had comparable specific activities of ≥18 GBq/μmol.

### Lipophilicity (log *D*_oct/PBS_) measurements

Lipophilicity of the employed compounds was determined by a conventional partition method between 1-octanol and phosphate buffered saline (PBS), pH 7.4. The 1-octanol was saturated with PBS before use. Briefly, the no-carrier-added tracers, contained in 0.2 mL PBS, were added 0.2 mL of 1-octanol in a 1.5-mL polypropylene Eppendorf vial. The vial was sealed and vigorously shaken at room temperature for 5 min. The mixture was then centrifuged at 3,000 *g* for 10 min. A 100 μL aliquot from each of the two phases was drawn and their radioactivity content was determined in a NaI (Tl) well-type detector. The log *D*_oct/PBS_ was calculated as follows: log *D*_oct/PBS_ = log (radioactivity concentration in the 1-octanol phase/radioactivity concentration in the PBS phase). The reported values represent the mean of six independent measurements.

### Brain uptake studies of FIBT, florbetaben, and PiB in BALB/c mice

*Ex vivo* brain uptake studies were performed in male BALB/c mice (mean ± SD body weight: 23 ± 2 g). Mice were injected via lateral tail vein with 4 to 6 MBq of high specific activity (≥11 GBq/μmol) [^18^F]FIBT, [^18^F]florbetaben, and [^11^C]PiB contained in 0.1 to 0.15 mL of a solution of isotonic phosphate buffered saline. Groups of mice were killed at 5 and 30 min p.i. Radioactivity of weighted brain samples was measured in a γ-counter (Wallac 1480-011 Automatic Gamma Counter, PerkinElmer, Waltham, MA, USA). Data are expressed as percent of the injected dose per gram tissue (% I.D./g; mean ± SD, *N* = 5).

### PET imaging

#### Small animal PET

Inhalation anesthesia with isoflurane was used and the eyes of the animals were protected with dexpanthenol eye ointment. Anesthesia was initiated 15 min ahead of experiments by placing the animal in a cage ventilated with oxygen (3.5 L/min) containing 3% isoflurane. Throughout the experiments, anesthesia was maintained by adjusting the isoflurane content (0.6% to 2%) to ensure a respiratory rate in the range 80 to 100/min. For i.v. injection, we inserted a tailored catheter into the lateral tail vein. Simultaneously with a slow bolus injection of 50 to 200 μL of tracer solution, we started the PET with scan duration of 45 min in 3D list mode on a Siemens Inveon system (axial field-of-view of 12.7 cm with a bore diameter of 12 cm, approximately 1.4 mm full-width-at-half-maximum spatial resolution; Siemens Healthcare, Erlangen, Germany). After tracer injection, we flushed the catheter with 50 to 100 μL of isotonic sodium chloride solution. During the scan, a heating pad prevented hypothermia. The radioactivity in the syringe was measured immediately before and after injection with a Capintec CRC® 15R (Capintec Inc, 6 Arrow Road Ramsey, NJ, USA) dose calibrator.

### Scanning regimen of PiB, FIBT, and florbetaben

Measurements of all three tracers in each animal were performed within a time period of 16 days to guarantee for comparable underlying neuropathology among the PET scans (Table 1).

**Table 1:** **Scanning regimen of [** ^**11**^ **C]PiB, [** ^**18**^ **F]FIBT, and [** ^**18**^ **F]florbetaben**

**Group**	**Number**	**Tracer 1**	**Injected dose (MBq)**	**Δ** ***t*** **to next scan (days)**	**Tracer 2**	**Injected dose (MBq)**	**Δ** ***t*** **to next scan (days)**	**Tracer 3**	**Injected dose (MBq)**
APP/PS1	1	Florbetaben	12.2	5	FIBT	9.4	3	PiB	8.9
	2	Florbetaben	8.9	5	FIBT	6.6	3	PiB	10.7
	3	Florbetaben	9.2	13	FIBT	16.3	3	PiB	19.5
	4	Florbetaben	13.4	13	FIBT	13.0	3	PiB	16.7
Ctl	1	Florbetaben	10.2	5	FIBT	10.6	3	PiB	14.3
	2	Florbetaben	7.9	5	FIBT	8.0	3	PiB	12.5
	3	Florbetaben	4.6	13	FIBT	14.0	3	PiB	18.4

### MR imaging of mice

A brain MR scan was performed on all tg and ctl mice within 0 to 6 days after PET scans. Throughout the MR scan, animals were under continuous 1.0% to 1.8% isoflurane anesthesia with 2 L/min oxygen flow. Eyes were protected with dexpanthenol ointment. Hypothermia was prevented with a heat storing gel pack (ColdHot, 3 M, Saint Paul, MN, USA) preheated in a microwave oven.

We acquired T1 weighted brain images using a 3D turbo gradient echo (3D-TFE) sequence with an inversion pre-pulse (TR: 12 ms, TE: 3.9 ms, TI: 800 ms, TFE factor: 120, flip angle: 8°, NSA: 12, acquired matrix M × P: 248 × 120, partitions: 60, FOV: 64 × 32 × 16 mm, resolution: 0.26 × 0.27 × 0.26 mm, reconstructed resolution: 0.13× 0.13 × 0.13 mm) on a Philips Achieva 1.5 T clinical MRI system with a 23-mm microscopy coil. Acquisition time was 46 min 11 s.

### Mouse PET and MRI

Based on our experience, variance of macroscopic brain anatomy is very low across mice with the same genetic background. Therefore, the creation of a MRI template was done by manual co-registration of all MR images based on prominent brain structures and the overall cortical contour and calculation of a mean image (Pmod 3.3, Pmod Technologies, Zurich, Switzerland). Structures outside of the brain were not considered in the manual co-registration process. Two major volume-of-interests (VOIs) were defined on the MRI template, one comprising the full cortex including hippocampus, the other comprising the full cerebellum. The same VOIs were then used for reading out co-registered PET data of a mean image of the final 10 min of the scan (minute 36 to 45). The VOIs were chosen based on previous findings in a voxel-based analysis of cerebral PiB uptake and its correlation with the underlying Aβ pathology in this mouse model [41]. PET data was normalized and corrected for decay and dead time. The images were reconstructed by means of filtered back-projection (FBP), using a ramp filter with a cutoff at the Nyquist frequency into 45 frames (45 × 60 s). The image volume consisted of 128 × 128 × 159 voxels, each of a size of 0.78 × 0.78 × 0.80 mm. The PET images were manually co-registered with the MRI template. This was done by using the wash-in phase of the tracer to identify the brain contour for alignment with the MRI template. We scaled the PET images to percentage of injected dose per cubic centimeter. We then applied these VOIs to the co-registered PET images and exported the VOI values to Microsoft Excel 2010 in order to calculate cortex + hippocampus-to-cerebellum ratios (further referred to as PET ratios).

We tested the tg/ctl ratios for significance. First, we performed a one-way ANOVA between the three groups showing significance. *Post hoc* Bonferroni corrected *t*-tests thresholded at *p* < 0.05 revealed that the significant difference is due to group differences between PiB and florbetaben and FIBT and florbetaben.

### Brain dissection and tissue section preparation

Immediately after the third PET scan, each animal was sacrificed per cervical dislocation. The skull was dissected and opened mid-sagittally with fine scissors. The brain was carefully removed, halved in the left and right hemisphere and frozen with fine crushed dry ice and stored in a refrigerator at −80°C. For histological staining, the right hemisphere of each animal was thawed, para-sagittal halved and paraffinated. The resulting paraffin blocks were then cut with a thickness of 5 μm and mounted 3 sections per glass slide.

### Thioflavin S staining and microscopy

Before staining, the sections were deparaffinated and rehydrated. The sections were then incubated in a 1% (1 g per 100 ml water) solution of Thioflavin S (TfS) (T1892, Sigma-Aldrich, St. Louis, MI, USA) for 30 min. The sections were then washed with water three times for 2 min, 80% ethanol for 6 min, washed again with water and cover slip mounted with VectaShield as mounting medium (H-1500 with DAPI, Vector Laboratories Inc., Burlingame, CA, USA). The slides were stored at 4°C until microscopic image acquisition. Digital images were acquired with an automated whole-slide-scanner (Mirax Scan, Carl Zeiss MicroImaging GmbH, Jena, Germany) using filter sets for DAPI, GFP, and Texas Red. The DAPI (contained in the mounting medium) fluorescence was used by the scanner to set the optical focus, GFP contained the specific signal of Thioflavin S, and Texas Red delivered unspecific fluorescence such as tissue auto-fluorescence. Images were pre-processed with Photoshop CS4 in order to define (blue-channel) the cortex and hippocampus as regions of interest for plaque load analysis.

### Histological plaque load analysis

To measure the relative plaque load in a region of interest (ROI) of each section, we used the Acapella^TM^ analysis software (PerkinElmer Inc., Waltham, MA, USA) applying a special script (developed by Evotec AG, Hamburg, Germany). This script allows quantitative assessment of the relative plaque load (sum of pixels representing plaque signals divided by the sum of all pixels representing the regions of interest). The Texas Red signal was used by Acapella^TM^ to correct the TfS signal for unspecific fluorescence.

Acapella analysis was verified on each section. Minor detection deficits were tolerated (e.g., failure to detect <5 plaques); larger deficits (e.g., failure to detect a group of plaques) lead to exclusion of the particular image. In cases of a large contamination detected as plaque-specific signal (e.g., a tissue fold or dust particle), the region of interest was adjusted. At least 8, maximal 12, sections per tg mouse per staining (respectively 3 to 6 sections per wild-type mouse) were analyzed and mean values of plaque load calculated per animal. Autoradiography (Additional file 1) was performed following procedures already reported [40].

### Human [^11^C]PiB PET

To enable a comparison to human [^11^C]PiB PET data, we chose randomly PET datasets from our database of AD patients and healthy controls at TUM. Subjects labelled as AD subjects in this database had a positive PiB scan together with measurable cognitive deficits in neuropsychological testing fulfilling the criteria of dementia. This data had been obtained on a SIEMENS ECAT HR+ scanner (CTI, Knoxville, TN, USA) according to an established and published protocol. Mean global cortical PiB uptake relative to cerebellum expressed as SUVR was calculated in 20 AD patients and in 15 healthy human control subjects using a previously established VOI-based approach [48,49].

## Results

### Comparison of fundamental characteristics

[^18^F]FIBT, [^18^F]florbetaben, and [^11^C]PiB were prepared with comparable high quality, purity, and specific activities of ≥18 GBq/μmol and directly introduced to a group of four APP/PS1 and three age-matched ctl mice. As lipophilicity is an important functional property of a ‘cerebral radiopharmaceutical,’ log D_oct/PBS_ were measured for [^18^F]FIBT, [^18^F]florbetaben, and [^11^C]PiB (Table 2). Lipophilicity of FIBT is slightly higher than those of florbetaben and PiB.

**Table 2:** **Log** ***D***_**oct/PBS**_ **values of tested compounds (mean ± SD;** ***N*** **= 6)**

**Tracer**	**[** ^**11**^ **C]PiB**	**[** ^**18**^ **F]FIBT**	**[** ^**18**^ **F]Florbetaben**
Log *D*_oct/PBS_	1.50 ± 0.11	1.92 ± 0.06	1.58 ± 0.13
*p*	[^18^F]FIBT > [^11^C]PiB*	[^18^F]FIBT > [^18^F]florbetaben*	[^18^F]Florbetaben > [^11^C]PiB

*Indicates significant difference at *p* < 0.05 in Bonferroni corrected *t*-tests.

**Figure 1:**
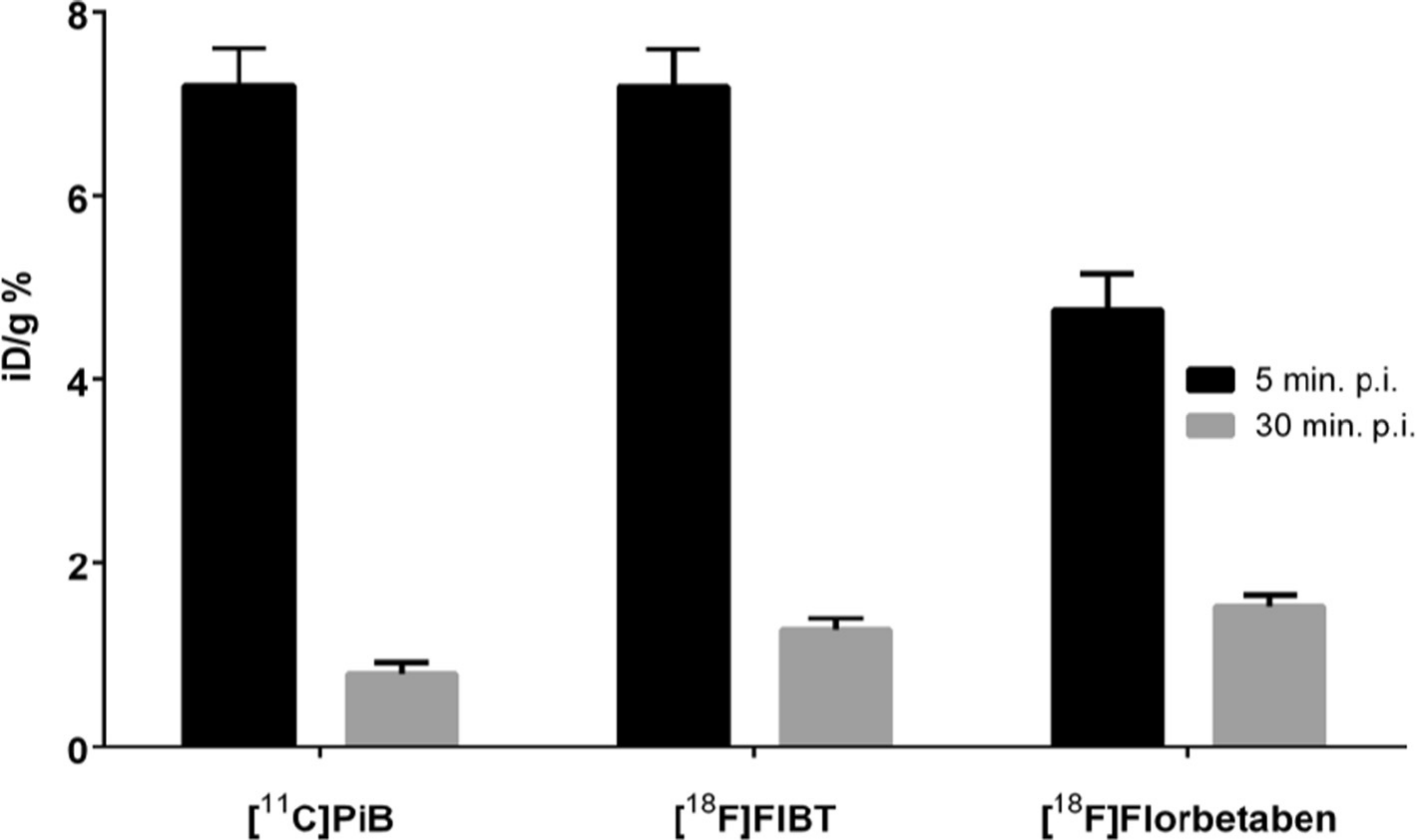
**Brain uptake of the tested radiopharmaceuticals.** Expressed as percent of injected dose per gram brain weight in male BALB/c mice at 5 and 30 min post injection (*N* = 5 for each tracer at each time point). p.i., post injection.

Brain uptake kinetics of florbetaben was measured at 5 and 30 min p.i. in wild-type mice (*N* ≥ 5) and compared to already published data for FIBT and PiB [25] (Figure 1).

### Intra-individual comparison of FIBT, florbetaben, and PiB in mice

**Figure 2:**
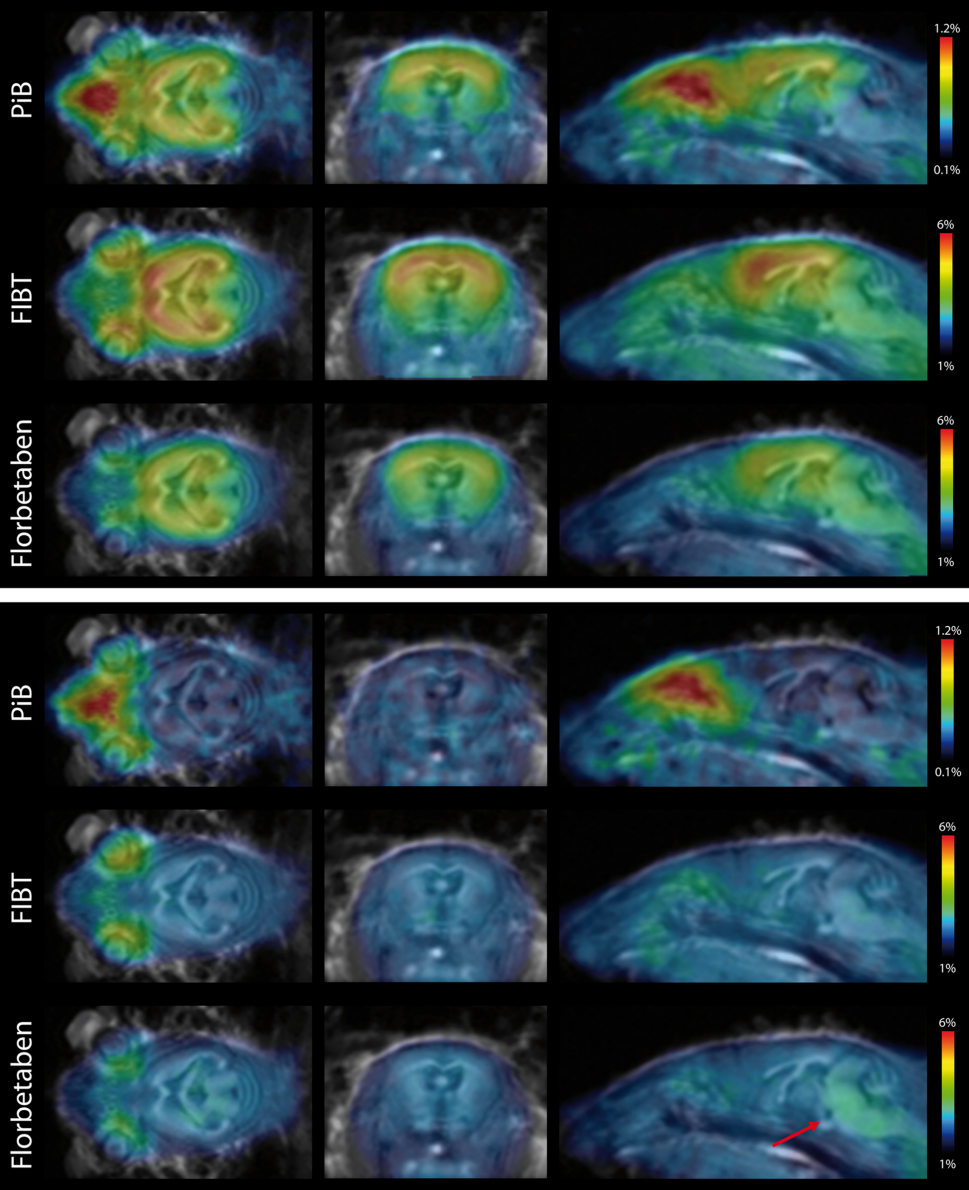
**Visual comparison of the three radiopharmaceuticals within the same group of animals.** Each row represents a mean image of four 24-month-old APP/PS1 transgenic animals (first three rows), respectively, three age matched wild-type animals (C57BL6/J, last three rows). *In vivo* PET images in each row represent percentage of injected dose per cubic centimeter (%ID/cc) averaged over 35 to 45 min post injection, co-registered to a MRI template. From left to right showing axial, sagittal, and coronal views. Red arrow points at higher unspecific uptake of florbetaben compared to FIBT in the brainstem of wild-type animals.

**Figure 3:**
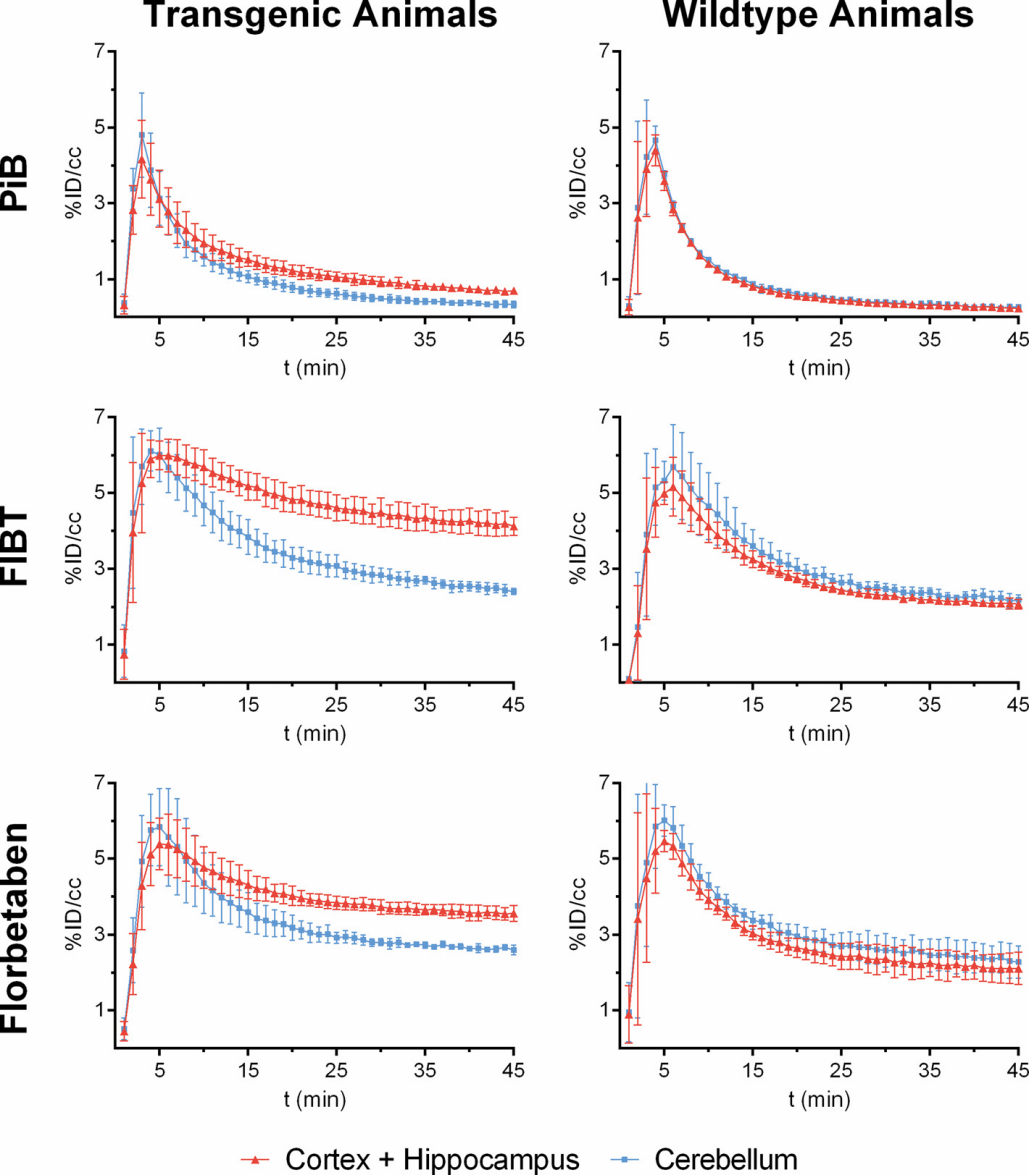
**Dynamic PET time activity curves of the cortex-VOI and the cerebellum-VOI.** Time-activity curves of the three radiopharmaceuticals (rows) within four APP/PS1 tg mice (left column) and three control mice (right column). Values are percentage of injected dose per cubic centimeter (%ID/cc) ± SD for a cortex + hippocampus VOI (red line) and a cerebellum VOI (blue line).

**Figure 4:**
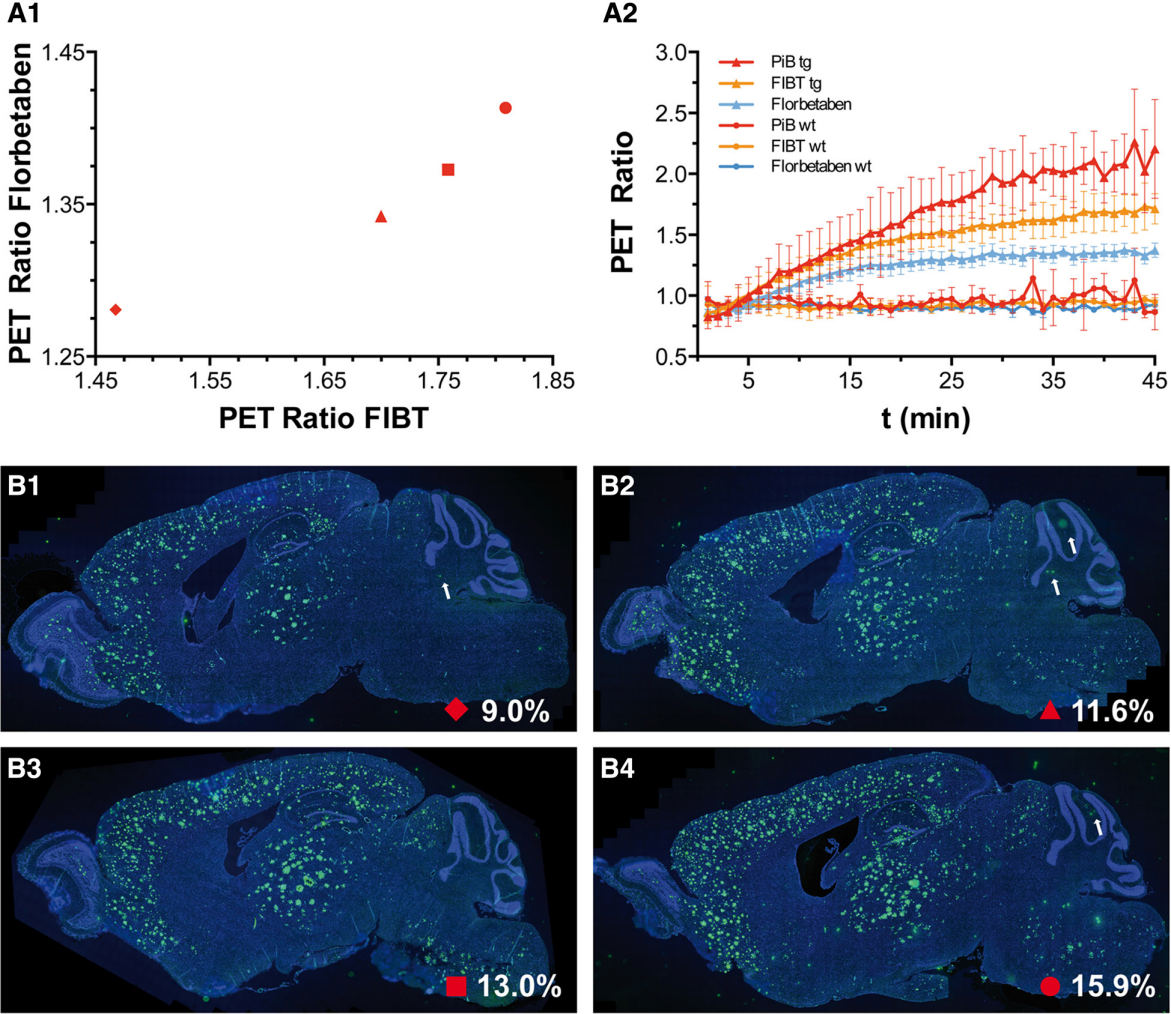
**PET ratios and group-wise mean dynamic PET ratio. (A1)** PET ratios within the same transgenic animals for FIBT (x-axis) and florbetaben (y-axis). **(A2)** Group-wise mean dynamic PET ratio curves for the three radiopharmaceuticals. The four different symbols in A1 mark the individual transgenic animal for which representative Thioflavin S staining images, and plaque load results are given in **(B1)** to **(B4)**. White arrows point at artefacts in the cerebellum that do not represent amyloid plaques.

*In vivo* small animal PET imaging of the three tracers was compared using a group of four APP/PS1 tg and three ctl mice, co-registered to the MRI template showing axial, sagittal, and coronal views of group-wise mean images (Figure 2). Dynamic PET time activity curves of the cortex-VOI and the cerebellum-VOI for FIBT, florbetaben, and PiB within identical mice are illustrated in Figure 3. The dynamic PET ratio curves are given in Figure 4, where tg and ctl mice showed significant differences in PET. [^11^C]PiB showed slightly lower initial uptake values than both ^18^F-tracers but considerably different wash-out. More pronounced [^11^C]PiB washout-out from Aβ-free regions was resulting in the highest target-to-reference-region ratios. The lower washout of [^18^F]florbetaben observed in BALB/c mice (Figure 1) was also observed on visual inspection of mean PET images of the control group. Here, a higher tracer retention of [^18^F]florbetaben compared to [^18^F]FIBT was detected in the brainstem (see red arrow in Figure 2). Furthermore, mean PET ratios (SUVR, calculated for time interval 36 to 45 min) for the APP/PS1 tg group and the control group as well as their ratio SUVR_tg_/SUVR_ctl_ are given in Table 3. Among the three tracers, [^11^C]PiB showed significantly higher unspecific nasal uptake in all cases. As observed for many PET tracers, all three tracers showed nonspecific uptake in the retrobulbar space, likely representing the Harderian gland.

**Table 3:** **Mean PET ratios (SUVR ± SD) within the four APP/PS1 tg mice and the three control mice (C57BL6/J)**

**Tracer**	**[** ^**18**^ **F]FIBT**	**[** ^**18**^ **F]Florbetaben**	**[** ^**11**^ **C]PiB**
APP/PS1 (*N* = 4)	1.68 ± 0.15	1.35 ± 0.06	2.08 ± 0.24
C57BL6/J (*N* = 3)	0.95 ± 0.02	0.90 ± 0.01	0.99 ± 0.06
tg/ctl	1.78 ± 0.16	1.50 ± 0.06	2.11 ± 0.24
*p*	[^18^F]FIBT > [^18^F]florbetaben*	[^11^C]PiB > [^18^F]florbetaben*	[^11^C]PiB > [^18^F]FIBT

The last row gives the statistical results of group comparisons of tg/ctl ratio values; *indicates significant difference at *p* < 0.05 in Bonferroni corrected *t*-tests.

### *Post mortem* plaque load analysis of APP/PS1 and ctl mice

**Figure 5:**
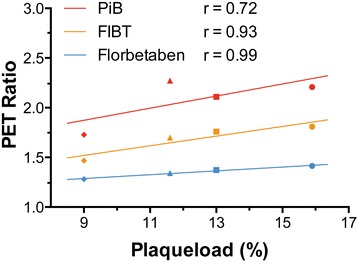
**Association of PET signal to Thioflavin S plaque load.** Lines represent linear regressions for each tracer. The four different symbols marking the individual transgenic animals are the same as used in Figure 4.

Brain sections of the animals were stained with Thioflavin S for the histological quantification of Aβ plaque load by applying a computerized image analysis and object recognition algorithm [33]. Significant Thioflavin S positive plaque load was found in each transgenic mouse (Figure 4B1 to B4). Mean false positive plaque load signal was found to be 0.03% (±0.01 SD) in the control group without amyloid pathology. We found a high linear association between plaque load and PET ratios for all three tracers (Figure 5).

### Human in relation to small-animal PiB PET

**Figure 6:**
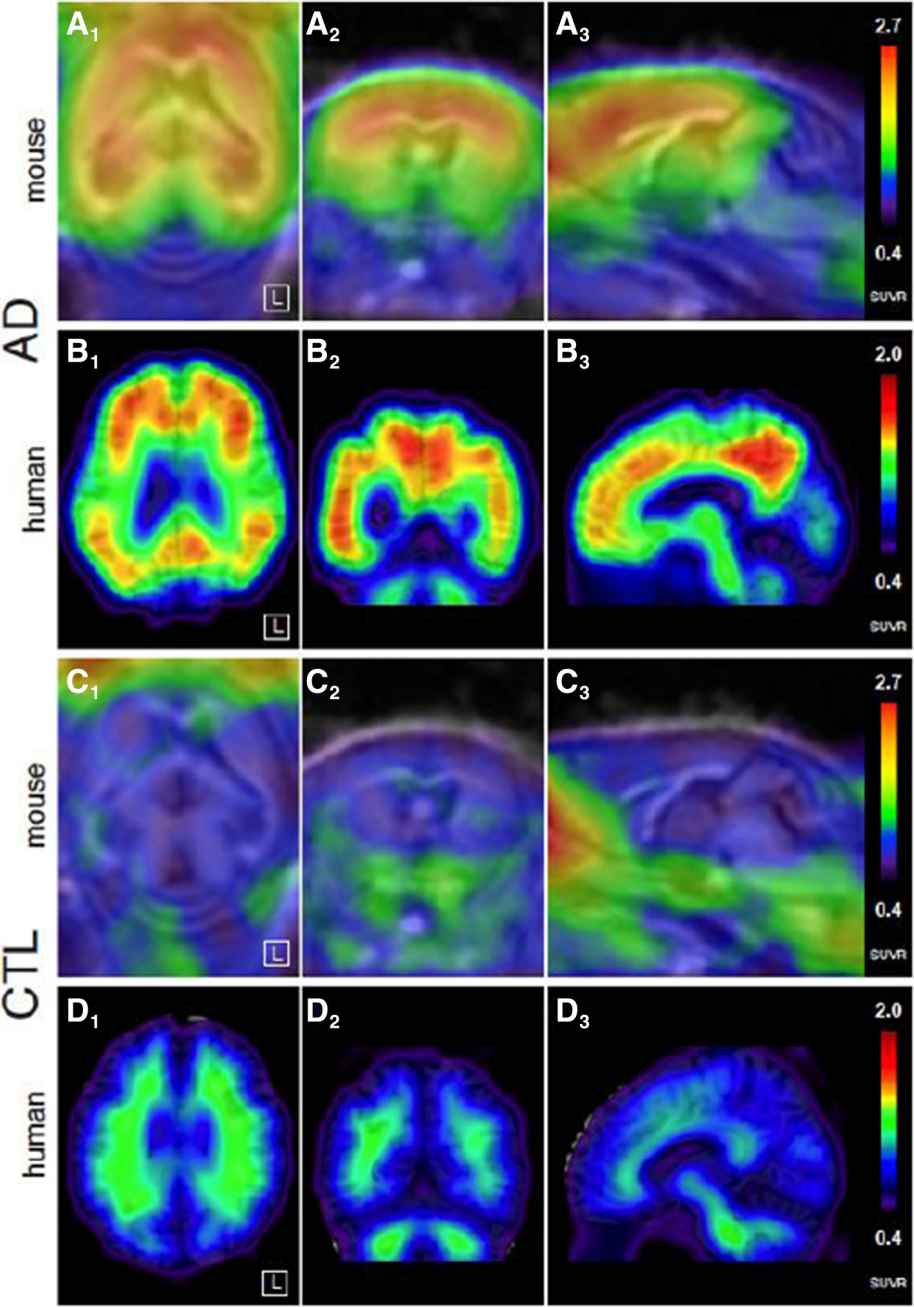
**Direct visual comparison of PiB PET in mice and human.** Image is showing from left to right axial, coronal, and sagittal views. Mouse images represent a mean image of the four APP/PS1 tg mice and the three control mice, respectively. Human images are from a representative AD patient and healthy control subject, respectively.

To find an expression for the translational value of the preclinical ranking of the three Aβ radiopharmaceuticals, a visual association of preclinical and clinical PiB data was laid out. Figure 6 illustrates the cortical tracer retention in mice in relation to humans. Analogous to AD patients, there is a strong uptake of [^11^C]PiB in cortical regions and unspecific low uptake in the cerebellum in transgenic mice. Human controls (HC) and control mice show only unspecific tracer retention across the whole brain.

*In vivo* PiB PET data in AD patients and human control subjects were also used for calculating SUVR (Table 4). The SUVR values for [^11^C]PiB of human AD patients are comparable to the [^11^C]PiB ratios found in our transgenic cohort (2.01 for AD patients vs. 2.08 in aged APP/PS1 tg mice).

**Table 4:** **Human in relation to mouse PET with [** ^**11**^ **C]PiB**

	**Human**	**Mouse**
AD	2.01 ± 0.29	2.08 ± 0.24
Controls	1.22 ± 0.12	0.99 ± 0.06
AD/ctl	1.65 ± 0.41	2.11 ± 0.24

Global cortex/cerebellum ratio (expressed as SUVR) in the human AD group (*N* = 20) and the HC group (*N* = 15) vs. four APP/PS1 tg mice and three control mice.

## Discussion

This study was motivated by the finding that despite a considerable number of ^18^F-labeled Aβ tracers that has been described, a final answer to the key question, which of these radiopharmaceuticals shows optimal properties, cannot be given on the basis of existing data. This is mainly due to the inhomogeneity of preclinical characterization methods applied, and further complicated by primarily ethical, but also financial and other practical restrictions of evaluating radiopharmaceuticals head-to-head in the same human subjects. In addition, it appears practically impossible to acquire enough preclinical data to enable a mutual comparison of every possible pair of tracers. As suggested previously [50], this situation could be circumvented by using [^11^C]PiB as common standard, which is recommendable due to the considerable body of knowledge already gained for this compound. Following this rationale, some of the upcoming ^18^F-labeled tracers have been compared to [^11^C]PiB [51,52]. Consequently, [^11^C]PiB PET results in mice and humans were also implemented in our study as an anchor point for comparison.

One previous paper compared two ^18^F-labeled Aβ tracers indirectly via two separate groups of AD patients with a [^11^C]PiB scan as link between the two groups [29]. However, this type of indirect comparison requires large cohorts to establish a valid comparison via statistical analysis. In terms of preclinical studies, an intra-individual based comparison of candidate tracers would require a direct correlation of the signal with the individual plaque load of the animal. This is necessary because one has to consider that in small animal models of AD, the inter-subject plaque load is generally not constant, which applies also to our transgenic model [33]. Hence, the most notable advantage of our intra-individual comparison approach is that it allows for drawing meaningful conclusions from smaller sample sizes (cohorts), thus resolving some ethical (cf. triple-R rule) and economical issues at the same time. Furthermore, a group of transgenic mice can, in principle, be used to compare even more than three Aβ tracers, which might prove a useful means of parallel Aβ tracer evaluation in future studies. Admittedly, a certain drawback of our protocol is that, in principle, absolute quantification of small animal PET signals requires complex image analysis steps. However, by calculating ROI ratios, we chose a straightforward approach, which is frequently applied in clinical as well as preclinical settings, and is widely considered to yield sufficiently precise data to enable meaningful conclusions. The linear association of PET ratios with plaque load for all three tracers in Figure 5 implies that calculating ROI ratios further supports this approach. With the possibility of subsequent *ex vivo* and histopathological analyses, the advantages of the preclinical approach presented here go far beyond merely circumventing challenges, such as ethical implications of repetitive scans in clinical studies.

We compared [^18^F]FIBT with [^18^F]florbetaben and [^11^C]PiB using small animal PET in aged transgenic APP/PS1 and control mice. The direct head-to-head PET comparison was supplemented by semi-automated histological plaque load analyses, using the brain material from the animals previously subjected to PET, and by *ex vivo* brain uptake kinetic analyses in a separate group of wild-type BALB/c mice. Pharmacokinetics of [^18^F]FIBT was superior to [^18^F]florbetaben which also resulted in better PET ratios (1.68 ± 0.15 versus 1.35 ± 0.06) in transgenic mice. However, PET ratios of both tracers correlated equally well with amyloid pathology.

Sufficient uptake in the brain and rapid wash-out of unbound tracer are important determinants of potential signal strength and sensitivity of a tracer. These pharmacokinetic properties are commonly assessed by *post mortem* determination of brain uptake kinetic analyses in healthy animals. At first glance, chemical properties of both ^18^F-labeled tracers meet the requirements for sufficient permeation of the blood-brain barrier, such as low molecular weight and medium lipophilicity [7]. However, we noted a lower initial uptake and higher tracer retention at 30 min p.i. of [^18^F]florbetaben compared to [^18^F]FIBT in animals without Aβ pathology (BALB/c mice). Higher initial uptake could be attributed to the slightly lower lipophilicity of [^18^F]florbetaben (log *D* = 1.58 ± 0.13) compared to [^18^F]FIBT (log *D* = 1.92 ± 0.06) [53,54] but might also be related to presence of hydrogen bond donors and acceptors as well as a higher percentage of polar surface area. Higher retention of [^18^F]florbetaben is likely due to a higher degree of unspecific binding. Hence, the low amount of unspecific retention of [^18^F]FIBT represents a substantial improvement, because compared to [^11^C]PiB, most ^18^F-labeled AD tracers in advanced clinical trials show a higher tendency towards unspecific binding to the white matter in humans [52,55].

Comparison of PET images of AD and control mice indicated higher unspecific binding of [^18^F]florbetaben compared to [^18^F]FIBT, especially in subcortical regions (red arrow in Figure 2), which is equivalent to a higher Aβ sensitivity of the latter. This might pose an important advantage with respect to the potential role of Aβ PET imaging in the pre-symptomatic phase of the disease [56], which is considered to begin several years before symptom onset (accumulation of pathological aggregates), and is likely the favorable time window for anti-amyloid interventions [57].

Apart from pharmacokinetic properties and specificity of binding, Aβ affinity and selectivity determines the quality of a tracer. In a competitive binding assay versus [^3^H]PiB, FIBT showed high affinity to Aβ aggregates (*K*_i_ = 2.1 ± 0.8 nM) [25]. Furthermore, *in vitro* determination of binding affinities to Aβ aggregates, using *post mortem* AD brain homogenates, suggest a higher Aβ affinity of [^18^F]FIBT (*K*_d_ = 0.7 ± 0.2 nM [28]) than [^18^F]florbetaben (*K*_d_ = 6.70 ± 0.30 nM, [16]). These results are nicely corroborated by our *in vivo* data, as we found higher tracer retention for [^18^F]FIBT than [^18^F]florbetaben in Aβ-rich brain regions of APP/PS1 transgenic mice (Figure 4A2). Since specific binding of PiB to Aβ in this mouse model was demonstrated previously by *in vitro*, *ex vivo*, and *in vivo* experiments [40], we conclude that higher retention of [^18^F]FIBT in target regions corresponds to improved specificity of binding *in vivo* as well.

Furthermore, it was shown earlier that the cortex-to-cerebellum ratios of PiB correlate well with the absolute amount of Aβ in the cortex, the place where most of the Aβ deposits are found [41]. Ratios of PiB in APP/PS1 transgenic mice closely resembled the ratios measured in our cohort of AD patients, further supporting the notion that our transgenic mouse model is suitable for PET imaging studies of Aβ pathology. Being aware that results from small animal experiments can usually not be directly translated to a clinical setting, we nevertheless hold the view that clinical properties of other tracers evaluated in the same setting could be predicted with certain accuracy. Thus, we assume that FIBT could perform very well in human as a promising diagnostic Aβ radiopharmaceutical and robust imaging tool with potential to become an earlier AD biomarker.

## Conclusions

With an increasing number of ^18^F-labeled Aβ tracers, comparative studies are of high value for providing diagnostic and prognostic information and feasibility rankings. Despite general problems of transferability of preclinical results to clinical settings, our study suggests that small animal PET imaging using a suitable animal model combined with *ex vivo* and *in vitro* experiments is a practicable approach for comparative Aβ tracer assessment. Overall, all assessed tracers allowed high-contrast imaging of Aβ deposits in transgenic models of AD. Our results indicate a superior performance of [^18^F]FIBT relative to [^18^F]florbetaben regarding pharmacokinetics and specific binding affinity towards Aβ aggregates. These results support further evaluation of [^18^F]FIBT in human investigations.

## Additional file

Additional file 1:
*Ex vivo* confirmation study. **Figure S1.** [^18^F]FIBT and [^18^F]florbetaben autoradiography and cortex/cerebellum uptake ratio (*n* ≥ 3).
