# Supplementary material for: FIBT versus florbetaben and PiB: a preclinical comparison study with amyloid-PET in transgenic mice
Source: EJNMMI Res. 2015 Mar 28;5:20. doi: 10.1186/s13550-015-0090-6 (PMC4402683; doi:10.1186/s13550-015-0090-6)
Supplement: Additional file 1: — Ex vivo confirmation study. Figure S1. [18F]FIBT and [18F]florbetaben autoradiography and cortex/cerebellum uptake ratio (n ≥ 3). [file 13550_2015_90_MOESM1_ESM.docx]

## Supporting Information FIBT^Ϯ^ versus Florbetaben and PiB: a Preclinical Comparison Study with Amyloid-PET in Transgenic Mice

Behrooz Hooshyar Yousefi^1*^, Boris von Reutern^2,4^, Daniela Scherübl^2^, André Manook^1^, Markus Schwaiger^2^, Timo Grimmer^3^, Gjermund Henriksen^2^, Stefan Förster^2^, Alexander Drzezga^2,5^ ,Hans-Jürgen Wester^1^

1) Department of Pharmaceutical Radiochemistry, Technische Universität München, Munich

2) Department of Nuclear Medicine, Technische Universität München, Munich

3) Department of Psychiatry and Psychotherapy, Technische Universität München, Munich

4) Institute of Neuroscience and Medicine (INM-3), Research Centre Jülich, Jülich

5) Department of Nuclear Medicine, University of Cologne, Cologne

### ^*^Corresponding author:

Behrooz H. Yousefi, PhD.

Technische Universität München

Department of Pharmaceutical Radiochemistry,

Walther-Meißner-Str. 3

85748 Garching

Germany

Tel: + 49 89 289 10265

Fax: + 49 89 289 12204

email: b.yousefi@tum.de

^Ϯ^2-(*p*-Methylaminophenyl)-7-(2-[^18^F]fluoroethoxy)imidazo[2,1-*b*]benzothiazole

### Ex vivo confirmation study

We have recently cross-validated the results from [^18^F]FIBT ex vivo autoradiography by co-injecting [^3^H]PiB combined with in vitro immunohistochemistry with specific antibodies for Aβ_40_ and Aβ_42_ and Thioflavin-S stains (Manook et al., 2012), which demonstrated a specific uptake of this PET-radiopharmaceutical to regions showing high concentrations of fibrillar amyloid-β in APP/PS1 tg mouse brain. In the present study, ex vivo autoradiography of [^18^F]FIBT and [^18^F]Florbetaben (Figure SI-1) were performed following a published procedure (Manook et al., 2012).

In accordance with the in vivo data (see Figure 2), a higher unspecific uptake in the white matter of control mice can be observed for [^18^F]Florbetaben compared to [^18^F]FIBT. Both Aβ radiopharmaceuticals show specific binding to Aβ deposits in the APP/PS1 tg mouse brain.


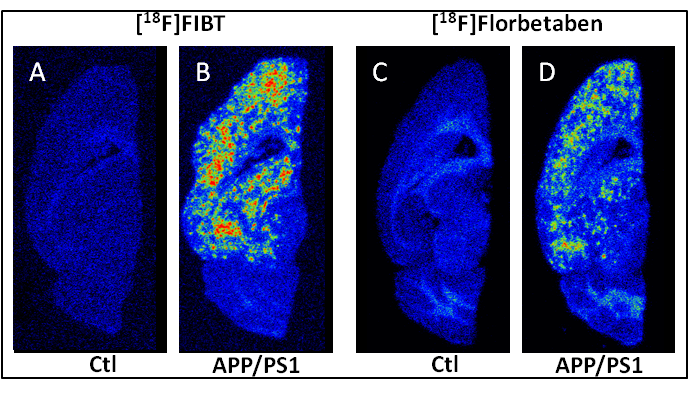


| [^18^F]FIBT | | | [^18^F]Florbetaben | |
| --- | --- | --- | --- | --- |
| Ctx/Clb ratio | Ctl | APP/PS1 | Ctl | APP/PS1 |
|  | 1.1 ± 0.1 | 4.7 ± 0.3 | 1.3 ± 0.1 | 2.0 ± 0.3 |

### Figure SI-1. [^18^F]FIBT and [^18^F]Florbetaben autoradiography and cortex/ cerebellum uptake ratio (n≥ 3). Images of digital ex vivo autoradiography of a 12 µm thick axial section of age- and gender matched control (A and C) and APP/PS1 tg mouse brain (B and D). [^18^F]FIBT (A and B), and [^18^F]Florbetaben (C and D).

### Reference

Manook A, Yousefi BH, Willuweit A, Platzer S, Reder S, Voss A et al. Small-animal PET imaging of amyloid-beta plaques with [^11^C]PiB and its multi-modal validation in an APP/PS1 mouse model of Alzheimer's disease. *PLoS One* **2012**;7(3):e31310. doi:10.1371/journal.pone.0031310.
